# Instant kit preparation of ^68^Ga-radiopharmaceuticals via the hybrid chelator DATA: clinical translation of [^68^Ga]Ga-DATA-TOC

**DOI:** 10.1186/s13550-019-0516-7

**Published:** 2019-05-23

**Authors:** Jean-Philippe Sinnes, Johannes Nagel, Bradley P. Waldron, Theodosia Maina, Berthold A. Nock, Ralf K. Bergmann, Martin Ullrich, Jens Pietzsch, Michael Bachmann, Richard P. Baum, Frank Rösch

**Affiliations:** 1 Institute of Nuclear Chemistry, Johannes Gutenberg-University Mainz, Mainz, Germany; 2 Molecular Radiopharmacy, INRASTES NCSR ‘Demokritos’, Athens, Greece; 3 Helmholtz-Zentrum Dresden-Rossendorf, Institute of Radiopharmaceutical Cancer Research, Dresden, Germany; 4 School of Science, Faculty of Chemistry and Food Chemistry, Technische Universität Dresden, Dresden, Germany; 5 Technische Universität Dresden, Universitätsklinikum ‘Carl Gustav Carus’, UniversitätsKrebsCentrum (UCC), Tumorimmunology, Dresden, Germany; 6 National Center for Tumor Diseases (NCT), Technische Universität Dresden, Dresden, Germany; 7 Zentralklinik Bad Berka GmbH, Clinic for Molecular Radiotherapy, Bad Berka, Germany

**Keywords:** Gallium-68, DATA-TOC, DOTA-TOC, NET, Somatostatin receptor, PET-CT, Molecular imaging

## Abstract

**Purpose:**

The widespread use of ^68^Ga for positron emission tomography (PET) relies on the development of radiopharmaceutical precursors that can be radiolabelled and dispensed in a simple, quick, and convenient manner. The DATA (6-amino-1,4-diazapine-triacetate) scaffold represents a novel hybrid chelator architecture possessing both cyclic and acyclic character that may allow for facile access to ^68^Ga-labelled tracers in the clinic. We report the first bifunctional DATA chelator conjugated to [Tyr^3^]octreotide (TOC), a somatostatin subtype 2 receptor (SST_2_)-targeting vector for imaging and functional characterisation of SSTR_2_ expressing tumours.

**Methods:**

The radiopharmaceutical precursor, DATA-TOC, was synthesised as previously described and used to complex ^nat^Ga(III) and ^68^Ga(III). Competition binding assays of [^nat^Ga]Ga-DATA-TOC or [^nat^Ga]Ga-DOTA-TOC against [^125^I-Tyr^25^]LTT-SS28 were conducted in membranes of HEK293 cells transfected to stably express one of the hSST_2,3,5_ receptor subtypes (HEK293-hSST_2/3/5_ cells). First in vivo studies were performed in female NMRI-nude mice bearing SST_2_-positive mouse phaeochromocytoma mCherry (MPC-mCherry) tumours to compare the in vivo SST_2_-specific tumour-targeting of [^68^Ga]Ga-DATA-TOC and its overall pharmacokinetics versus the [^68^Ga]Ga-DOTA-TOC reference. A direct comparison of [^68^Ga]Ga-DATA-TOC with the well-established PET radiotracer [^68^Ga]Ga-DOTA-TOC was additionally performed in a 46-year-old male patient with a well-differentiated NET (neuroendocrine tumour), representing the first in human administration of [^68^Ga]Ga-DATA-TOC.

**Results:**

DATA-TOC was labelled with ^68^Ga with a radiolabelling efficiency of > 95% in less than 10 min at ambient temperature. A molar activity up to 35 MBq/nmol was achieved. The hSST_2_-affinities of [^nat^Ga]Ga-DATA-TOC and [^nat^Ga]Ga-DOTA-TOC were found similar with only sub-nanomolar differences in the respective IC_50_ values. In mice, [^68^Ga]Ga-DATA-TOC was able to visualise the tumour lesions, showing standardised uptake values (SUVs) similar to [^68^Ga]Ga-DOTA-TOC. Direct comparison of the two PET tracers in a NET patient revealed very similar tumour uptake for the two ^68^Ga-radiotracers, but with a higher tumour-to-liver contrast for [^68^Ga]Ga-DATA-TOC.

**Conclusion:**

[^68^Ga]Ga-DATA-TOC was prepared, to a quality appropriate for in vivo use, following a highly efficient kit type process. Furthermore, the novel radiopharmaceutical was comparable or better than [^68^Ga]Ga-DOTA-TOC in all preclinical tests, achieving a higher tumour-to-liver contrast in a NET-patient. The results illustrate the potential of the DATA-chelator to facilitate the access to and preparation of ^68^Ga-radiotracers in a routine clinical radiopharmacy setting.

**Electronic supplementary material:**

The online version of this article (10.1186/s13550-019-0516-7) contains supplementary material, which is available to authorized users.

## Background

There has been a surge in the development of ^68^Ga-radiopharmaceuticals over the last decade initiated by the clinical and commercial success of [^68^Ga]Ga-DOTA-TOC (TOC: DPhe-c[Cys-Tyr-DTrp-Lys-Thr-Cys]-Thr-ol) and [^68^Ga]Ga-DOTA-TATE (TATE: DPhe-c[Cys-Tyr-DTrp-Lys-Thr-Cys]-Thr-OH), as well as by significant improvements in the provision of ^68^Ga generators now fulfilling pharmaceutical standards [1–6].

As a result, [^68^Ga]Ga-DOTA-TOC and [^68^Ga]Ga-DOTA-TATE are currently being used in clinical settings for the diagnosis of neuroendocrine tumours (NETs). Furthermore, [^68^Ga]Ga-DOTA-TATE acquired FDA approval as a diagnostic PET radiopharmaceutical for the visualisation of NET lesions (FDA News Release, June 1, 2016), following the ‘orphan drug’ designation to [^68^Ga]Ga-DOTA-TOC by FDA [7], and [^68^Ga]Ga-DOTA-TOC was approved by European Medicines Agency. Due to the availability of ^68^Ga via commercial ^68^Ge/^68^Ga generators and favourable emission characteristics for PET imaging (*β*^+^ = 89%, *E*_β,max_ = 1.9 MeV), the facile and efficient access to ^68^Ga-radiopharmaceuticals is expected to drive the use of ^68^Ga in PET centres [1, 8–11]. A key step in this direction is the development of simple, effective, robust, and reliable labelling protocols, which depend primarily on the chelating moiety of the bifunctional chelator (BFC) attached to the vector of interest. Established BFCs based on a DOTA or DO3A scaffold for ^68^Ga require relatively harsh conditions (a balance of high temperatures, low pH, and high concentrations of the precursor) for efficient radiolabelling [2, 12]. This restriction inherently limits the portfolio of ^68^Ga-radiopharmaceuticals, because several promising peptide-, protein-, and antibody-based vectors for application in nuclear oncology are temperature and/or pH sensitive [13]. Thus, the radiolabelling of such molecules imposes stringent requirements on the BFC, i.e. > 95% labelling efficiency at ambient temperatures, less acidic conditions, and at high molar activities. Moreover, in the case of short-lived radionuclides, like ^68^Ga (*t*_1/2_ = 67.7 min), shorter labelling times and simple preparations are highly desirable, leading to a ready-for-injection radiolabelled product that does not require further purification prior to use. The development of such labelling protocols should be seen as mandatory to fully exploit the aforementioned advantages of ^68^Ga, but presents significant challenges in the design of suitable BFCs [14].

In general, chelators (Additional file 1: Figure S1) can be distinguished as cyclic (DOTA, NOTA, TRAP), associated with high thermodynamic stability, or acyclic (DFO, DTPA, HBED, THP), linked to a high kinetic stability that allows for higher labelling efficiency [12, 15–19]. For example, Blower et al. demonstrated that THP derivatives are superior in terms of labelling kinetics [20] compared to BFCs with cyclic chelating functionalities and the novel THP-conjugated radiopharmaceuticals are under evaluation to prove their full viability in vivo for different targeting vectors.

**Fig. 1:**
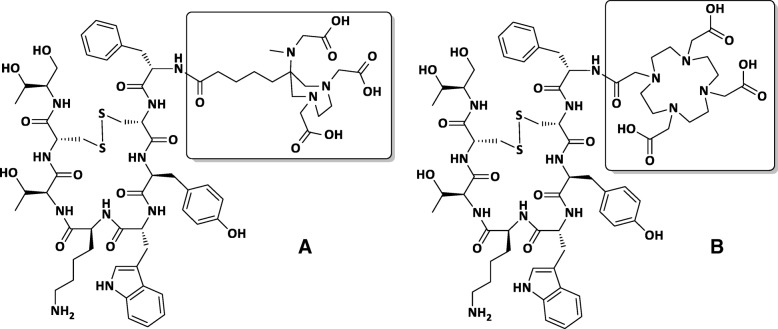
Structures of TOC coupled with **a** DATA and **b** DOTA. Chelator structures are highlighted

Special chelators like TRAP offer good properties in general, but are predominantly seen in the context of multivalent applications. The DATA scaffold represents a unique approach to chelator design in that the chelating moiety is a hybrid, possessing both cyclic and acyclic character. It is believed that flexibility of the acyclic portion (6′ nitrogen and associated acetate function) facilitates rapid complexation, whilst the preorganised cyclic portion minimises the energy barrier to complexation and inhibits decomplexation processes [21, 22]. The favourable radiolabelling kinetics of the DATA chelators, ambient temperature, and pH 4–6.5, along with the excellent stability of the forming ^68^Ga-chelates, justified the development of a bifunctional derivative [23]. We recently reported on the synthesis and ^68^Ga-radiolabelling of the first DATA peptide conjugate, DATA-TOC (Fig. 1) [23].

Following the promising results of the initial work with uncoupled DATA-BFCs, the aim was to evaluate the suitability of a DATA-BFC with an established vector for comparison with the current clinical standard. Therefore, [^68^Ga]Ga-DATA-TOC was selected as the first target for comparison with [^68^Ga]Ga-DOTA-TOC as the clinically established reference in a series of biological in vitro and in vivo models expressing the somatostatin subtype 2 receptor (hSST_2_), specifically (i) competition binding assays in human SST_2/3/5_-positive cell membranes, (ii) biodistribution and small animal PET imaging in a preclinical mCherry-expressing mouse phaeochromocytoma (MPC-mCherry) model with high SST_2_ density [24, 25], and (iii) clinical studies in a patient previously diagnosed with NETs. This direct comparison will reveal the influence of a DOTA-to-DATA chelator-switch on the biological behaviour of ^68^Ga-labelled DOTA-TOC.

## Materials and methods

DATA-TOC was synthesised as previously described [23], whilst DOTA-TOC was purchased from ABX GmbH. The ^nat^Ga complexes, [^nat^Ga]Ga-DATA-TOC and [^nat^Ga]Ga-DOTA-TOC, were obtained after treatment of the respective peptide conjugates with an excess of ^nat^GaCl_3_ and subsequently purified by HPLC (Luna 10 μm (C18) 100 Å (250 mm × 10 mm, 10 μm); A: H_2_O, B: MeCN). The retention time (*t*_R_) of [^nat^Ga]Ga-DOTA-TOC and [^nat^Ga]Ga-DATA-TOC is 18.6 min and 19.9 min, respectively (linear gradient: 5% MeCN to 50% MeCN in 20 min).

LTT-SS28 (H-Ser-Ala-Asn-Ser-Asn-Pro-Ala-Leu-Ala-Pro-Arg-Glu-Arg-Lys-Ala-Gly-c[Cys-Lys-Asn-Phe-Phe-DTrp-Lys-Thr-Tyr-Thr-Ser-Cys]-OH) was purchased from Bachem.

Gallium-68 was eluted from one of two available ^68^Ge/^68^Ga generators (iThemba Labs) using 1.0 M HCl. The final HCl concentration of the eluates from both generators was approximately 1 M. The pH of the fractionated ^68^Ga-eluate (300 μL containing 555 MBq (15 mCi) ^68^Ga at the start of synthesis) was adjusted to pH 4.0–4.5 using 2.0 M NH_4_OAc. A solution of ^68^Ga(OAc)_3_ in acetate buffer was added to 20 nmol of each peptide. The reaction mixture was shaken for 10 min at 80 °C to afford [^68^Ga]Ga-DOTA-TOC or at 20 °C for 10 min to afford [^68^Ga]Ga-DATA-TOC. Reaction mixtures were analysed by radio-HPLC.

Radio-HPLC was performed on a Series 1200 (Agilent) HPLC equipped with the Ramona ß/γ-ray radiodetector (Raytest): eluent A 0.1% (*v*/*v*) TFA in H_2_O; eluent B 0.1% (*v*/*v*) TFA in MeCN; HPLC system Zorbax (Agilent) SB-C18, 300 Å, 4 μm, 250 mm × 9.4 mm; and linear gradient elution using 95% eluent A to 95% eluent B in 10 min, 50 °C. Radiolabelled products with radiochemical purity higher than 95% were used for biological experiments after filtering (45 μm pore size, REZIST 13/0.45 PTFE, Schleicher & Schuell) and diluting the labelling reaction mixture. Filtrates were diluted with 0.1 mL electrolyte solution E-153 (Serumwerk Bernburg AG) to a final concentration of ~ 80 MBq/mL [26, 27].

For the preparation of [^125^I-Tyr^25^]LTT-SS28, [^125^I]NaI was provided by PerkinElmer in dilute sodium hydroxide solution (pH 8–11) in an activity concentration of 13.52 GBq (365.4 mCi) per mL. Radioiodination was performed according to the chloramine-T method using 0.1 M d,l-methionine to quench the reaction, and the radioligand was isolated by HPLC, as previously described [28–30].

### Cell culture and in vitro assays

The HEK293 cell line was transfected to stably express each of the hSST_2/3/5_ and the resultant HEK293-hSST_2/3/5_ cells used for receptor affinity assessments were donated by Prof. S. Schultz (Institute of Pharmacology and Toxicology, University Hospital, Friedrich Schiller University Jena, Germany). Cells were cultured at 37 °C and 5% CO_2_ in Dulbecco’s modified Eagle’s medium containing 10% fetal bovine serum, 100 U/mL penicillin, 100 mg/mL streptomycin and 500 mg/mL G418, as previously described [28, 30]. Culture reagents were from Gibco BRL, Life Technologies, and Biochrom KG Seromed.

The genetically modified MPC-mCherry cells [25] derived from MPC cells (clone 4/30PRR [31]) characterised by a high density expression of mouse SST_2_ [24] were cultured and prepared for in vivo application as previously described [24, 25].

Competition binding experiments were performed for [^nat^Ga]Ga-DATA-TOC and [^nat^Ga]Ga-DOTA-TOC in HEK293-hSST_2/3/5_ cell membranes, harvested as previously described [29]. [^125^I-Tyr^25^]LTT-SS28 served as radioligand and LTT-SS28 ([Leu^8^,DTrp^22^,Tyr^25^]SS28) as reference compound [28, 30, 32]. Briefly, radioligand (70 μL, 50 pM corresponding to ≈ 40,000 cpm), test peptide (30 μL solution of increasing concentrations, 10^− 5^–10^− 13^ M), and membrane homogenates (200 μL) were added to each assay tube (total volume of 300 μL in binding solution: 50 mM HEPES pH 7.4, 1% BSA, 5.5 mM MgCl_2_, 35 μM bacitracin) in triplicates for each concentration point. Samples were incubated for 60 min at 22 °C in an Incubator-Orbital Shaker unit (MPM Instr. SrI). Ice-cold washing buffer (10 mM HEPES pH 7.4, 150 mM NaCl) was added, followed by rapid filtration using glass fibre filters (Whatman GF/B, presoaked for 2 h in a 1% polyethyleneimine (PEI) aqueous solution) on a Brandel Cell Harvester (Adi Hassel Ingenieur Büro) washed with ice-cold washing buffer. Filters were collected, and their activity measured in a γ-counter (automated well-type multisample gamma counter; NaI(Tl) 3″ crystal, Canberra Packard Auto-Gamma 5000 series instrument). The half maximal inhibitory concentration (IC_50_) values were calculated by nonlinear regression according to a one-site model applying the PRISM 2 program (Graph Pad Software) and represent mean IC_50_ ± sd from *n* experiments performed in triplicate for [^nat^Ga]Ga-DATA-TOC (*n* = 3), [^nat^Ga]Ga-DOTA-TOC (*n* = 2), and LTT-SS28 (*n* = 3).

### Animal studies

A number of 2 × 10^6^ MPC-mCherry cells were transplanted subcutaneously into the right shoulder of female NMRI-nude mice (8 to 10 weeks old, RjOrl:NMRI-Foxn1^nu^ /Foxn1^nu^, Janvier Labs). Tumour growth was monitored by fluorescence imaging using the in vivo Xtreme optical imaging system (Bruker) [24] under anaesthesia that was induced and maintained by inhalation of 12% and 9% (*v*/*v*) desflurane in 30/10% (*v*/*v*) oxygen/air, respectively. Animals were studied when the tumour diameter was 6 to 9 mm.

For biodistribution studies with [^68^Ga]Ga-DATA-TOC 17 (control *n* = 9, blocked *n* = 8) and [^68^Ga]Ga-DOTA-TOC 12 (control *n* = 5, blocked *n* = 7) female mice (body weight 36.3 ± 2.1 g) were injected intravenously into a tail vein with approximately 2.3 MBq (62 μCi)/0.35 nmol peptide (DATA-TOC 11.2 nmol/kg body weight and DOTA-TOC 10.5 nmol/kg body weight) in 0.1 mL electrolyte solution E-153 (Serumwerk Bernburg AG) without (control) or with simultaneous injection of 100 μg/mouse [Nal^3^]octreotide acetate (blocked). Animals were sacrificed at 60 min post-injection (p.i.). Blood, tumour, and the major organs were collected, weighed, and counted in a cross-calibrated γ-counter (Isomed 1000, Isomed GmbH) and Wallac WIZARD Automatic Gamma Counter (PerkinElmer). The activity of the tissue samples was decay-corrected and calibrated by comparing the counts in tissue with the counts in aliquots of the injected radiotracer that had been measured in the γ-counter at the same time. The activity in the selected organs was expressed as percent-injected activity per organ (%IA) and the activity concentration in tissues and organs as standardised uptake value (SUV in [MBq activity/g tissue]/[MBq injected activity/g body weight]). Values are quoted as mean ± standard deviation for each group of animals.

PET scans were performed using a dedicated rodent PET/CT scanner (NanoPET/CT, Mediso). Anaesthetised mice (two animals per group) bearing subcutaneous MPC-mCherry-tumours on the right shoulder were positioned on a warmed bed along the scanner axis. The ^68^Ga-labelled product, 10 MBq/0.26 nmol/300 μL (8.6 nmol DATA-TOC/kg body weight) and 10 MBq/0.26 nmol/300 μL (14.1 nmol DOTA-TOC/kg body weight), was infused over 1 min into a tail vein. PET images were acquired beginning with the injection on a Mediso NanoPET/CT camera and were reconstructed in dynamic mode with 38 frames and 0.5 mm^3^ voxel size. Total scan time was 2 h. Region-of-interest (ROI) quantification was performed with ROVER (ABX GmbH). The ROI values were not corrected for recovery and partial volume effects. For each nanoPET/CT scan, 3D ROIs were drawn over the tumour, heart, muscle, liver, and kidneys in decay-corrected whole-body orthogonal images.

Statistical analyses were carried out with GraphPad Prism version 6 (GraphPad Software). The data expressed as mean ± SEM was submitted to a one-way analysis of variance (ANOVA) with post hoc Tukey’s multiple comparisons test, with a single pooled variance. Values of *P* < 0.05 were considered statistically significant and indicated by an asterisk (*).

### Human studies

A direct comparison between [^68^Ga]Ga-DATA-TOC and [^68^Ga]Ga-DOTA-TOC was performed in a 46-year-old male patient with well-differentiated NET lesions in the body and tail of the pancreas as well as peritumoural lymph node metastases, first diagnosed in November 2012. The large primary tumour involving the stomach, the spleen, and the left adrenal gland was surgically resected (R2) by distal pancreatectomy, partial gastrectomy, splenectomy, left adrenalectomy, and omentectomy. Despite octreotide therapy, the disease was progressing, and in 2015, the patient was treated with peptide receptor radionuclide therapy (PRRT), administering 5 GBq of [^90^Y]Y-DOTA-TOC. Before the second cycle, restaging was performed on a Biograph mCT FLOW 64 PET/CT from the vertex until mid-thigh exactly 50 min after separate injections of 117 MBq of [^68^Ga]Ga-DOTA-TOC (~ 5 μg peptide) and 120 MBq of [^68^Ga]Ga-DATA-TOC (~ 5 μg peptide). The PET/CT with [^68^Ga]Ga-DATA-TOC was performed 24 h after the [^68^Ga]Ga-DOTA-TOC PET/CT. Written informed consent was obtained from the patient in accordance with paragraph 37 of the updated Declaration of Helsinki, ‘Unproven Interventions in Clinical Practice’, and in accordance with the German Medical Products Act AMG §13 2b.

## Results

### Affinity of [^nat^Ga]Ga-DATA-TOC and [^nat^Ga]Ga-DOTA-TOC for the hSST_2/3/5_

**Fig. 2:**
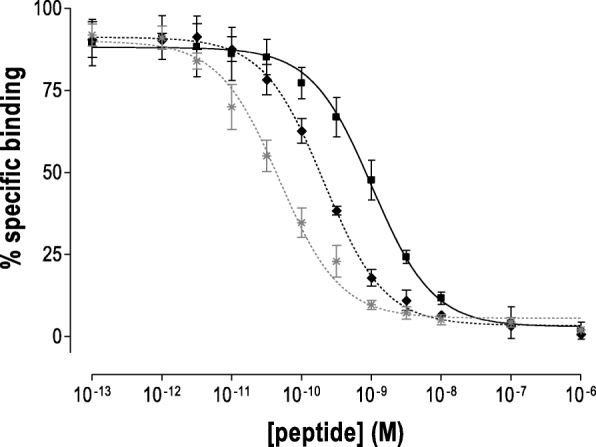
Displacement of [^125^I-Tyr^25^]LTT-SS28 from hSST_2_ binding sites in HEK293-hSST_2_ cell membranes by increasing concentrations of the following: square, [^nat^Ga]Ga-DATA-TOC (IC_50_ = 1.03 ± 0.08 nM, *n* = 3); diamond, [^nat^Ga]Ga-DOTA-TOC (IC_50_ = 0.21 ± 0.01 nM, *n* = 2); and control: asterisk, LTT-SS28 (IC_50_ = 0.05 ± 0.01 nM, *n* = 3). Results represent the average IC_50_ values ± sd of independent experiments performed in triplicate

The metallated peptide conjugates [^nat^Ga]Ga-DATA-TOC and [^nat^Ga]Ga-DOTA-TOC were tested for their ability to displace the pansomatostatin radioligand [^125^I-Tyr^25^]LTT-SS28 from hSST_2/3/5_-binding sites in HEK293-hSST_2/3/5_ cell membranes using the pansomatostatin ligand LTT-SS28 as reference [28–30]. As shown in Fig. 2, both metallated octapeptide analogs exhibited high affinity for the hSST_2_ ([^nat^Ga]Ga-DATA-TOC, IC_50_ = 1.03 ± 0.08 nM, and [^nat^Ga]Ga-DOTA-TOC, IC_50_ = 0.21 ± 0.01 nM). Compared to LTT-SS28 (IC_50_ = 0.05 ± 0.01 nM), both analogs were less affine, but absolute differences in the respective IC_50_ values were rather small [32]. It should be noted, that LTT-SS28 displayed sub-nM affinity also for hSST_3_ (IC_50_ = 0.9 ± 0.01 nM) and hSST_5_ (IC_50_ = 0.17 ± 0.03 nM). In contrast, [^nat^Ga]Ga-DATA-TOC and [^nat^Ga]Ga-DOTA-TOC were found to be hSST_2_ preferring (Additional file 1: Table S1).

### Small animal PET and biodistribution

#### Micro-PET imaging: specific tumour binding

**Fig. 3:**
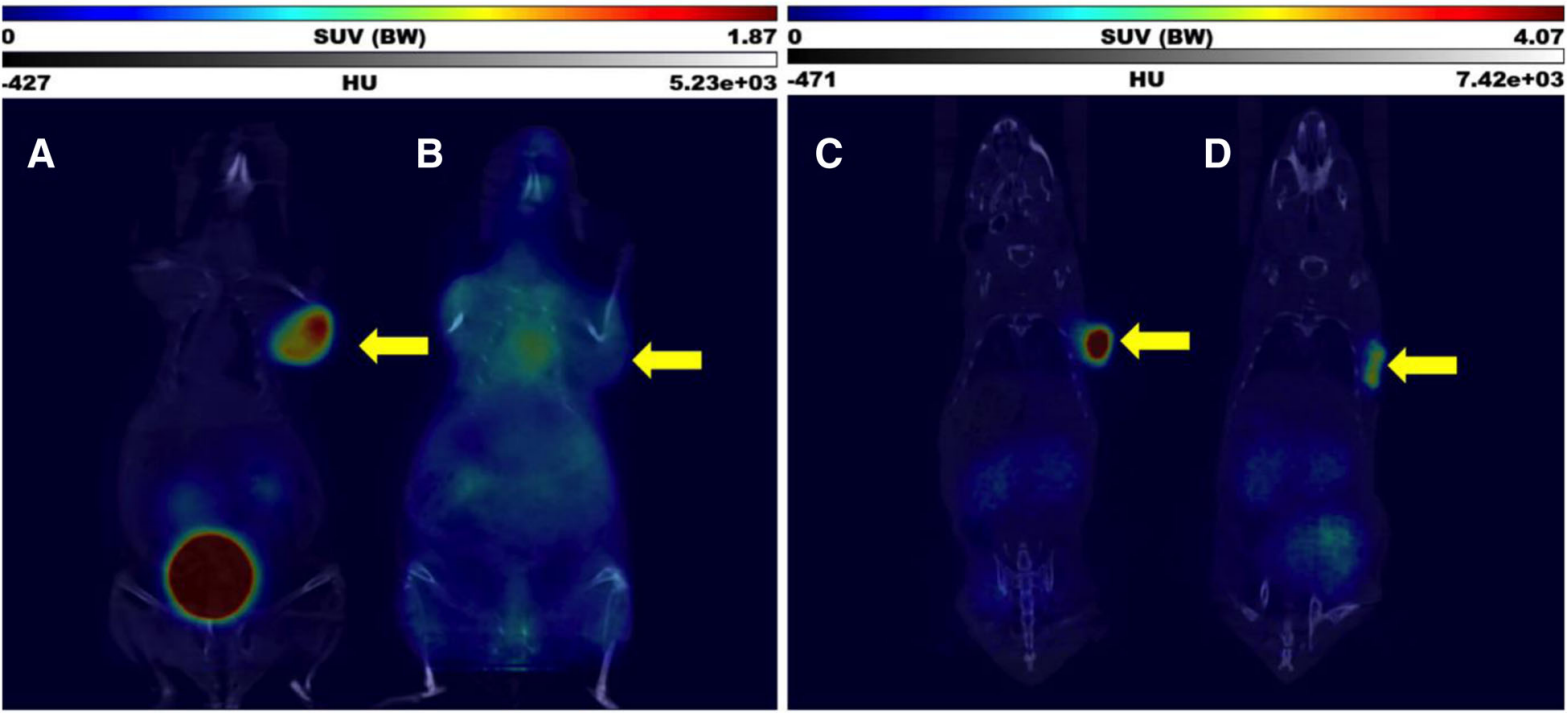
Coronal sections of PET images summarised from 1 to 2 h p.i. (midframe time 90 min), from dynamic PET studies of [^68^Ga]Ga-DATA-TOC (A, B) and [^68^Ga]Ga-DOTA-TOC (C, D) distribution in MPC-mCherry tumour-bearing NMRI nu/nu mice after single intravenous injections of the radiotracers as control (A, C) and blocked (B, D) by 100 μg/mouse [Nal^3^]octreotide acetate. The yellow arrows show the positions of the tumours

In dynamic PET studies in NMRI-nude mice, the implanted allogenic subcutaneous MPC-mCherry tumour was clearly visible with both radiotracers. Figure 3 shows coronal sections of dynamic PET images summarised from 1 to 2 h p.i. (midframe time 90 min) for one animal each. In vivo data for [^68^Ga]Ga-DATA-TOC and [^68^Ga]Ga-DOTA-TOC are illustrated for one animal each under A and C, respectively. For both radiotracers, the micro-PET data show a high accumulation of the radiotracers in the tumours. On a quantitative scale, the SUV (given in Fig. 3) appears to be higher for [^68^Ga]Ga-DOTA-TOC. However, the micro-PET data are affected by photon energies of ^68^Ga, by partial volume and spill-over effects. Accordingly, for a quantitative and statistically relevant comparison, we performed ex vivo organ distributions with *n* = 9 animals, see below.

In addition to the absolute tumour uptake of the two tracers it is important to address the specificity of the binding. Figure 3 shows the in vivo data for [^68^Ga]Ga-DATA-TOC and [^68^Ga]Ga-DOTA-TOC for the same animal with the SST_2_ blocked by 100 μg/mouse [Nal^3^]octreotide acetate, charts B and D, respectively. Qualitatively, both [^68^Ga]Ga-DATA-TOC and [^68^Ga]Ga-DOTA-TOC demonstrated specific binding to the tumours which could be blocked effectively. The quantitative degree of the blocking study again was addressed by ex vivo organ distribution studies.

Concerning the kidneys visualised in Fig. 3, kidney uptake is dependent in part on the individual hydration, urine flow of the mouse, and level of the anaesthesia. The figure shows individual mice at a specific time point during the PET study. The accumulation of the radiotracers in the kidney may differ across mice and from timepoint to timepoint. Consequently, kidney uptake is also addressed in the ex vivo biodistribution studies.

#### Micro-PET imaging: kinetics of tumour binding

**Fig. 4:**
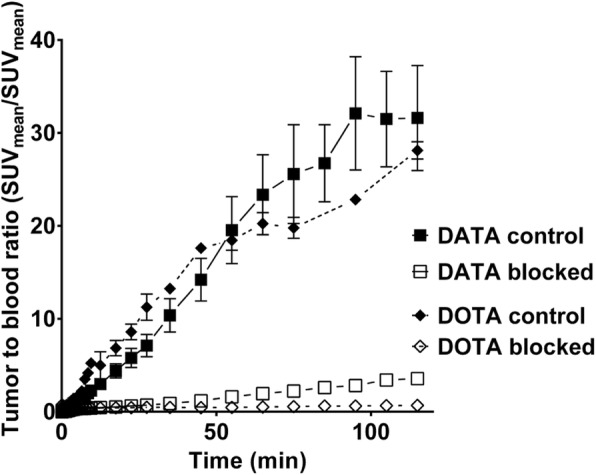
Tumour-to-blood ratios of [^68^Ga]Ga-DATA-TOC and [^68^Ga]Ga-DOTA-TOC calculated from dynamic PET studies with two animals per group (values are mean ± SEM)

The in vivo PET studies allow kinetic data for the SUV in several organs at different timepoints p.i. to be collected. Figure 4 shows the ratios between tumour and blood as SUV_mean_ (tumour)/SUV_mean_ (blood). The kinetic tumour-to-blood ratios of [^68^Ga]Ga-DATA-TOC and [^68^Ga]Ga-DOTA-TOC show a similar linear shape. At 1 h p.i., the tumour-to-blood ratios (standard uptake ratio, SUR) of the control experiments with both compounds reached similar levels of 31.6 ± 16.0 (*n* = 2) and 28.1 ± 1.3 (*n* = 3) for [^68^Ga]Ga-DATA-TOC and [^68^Ga]Ga-DOTA-TOC, respectively. SUR values for the blocking study are 3.6 ± 0.0 (*n* = 2) and 2.7 ± 0.3 (*n* = 2) for [^68^Ga]Ga-DATA-TOC and [^68^Ga]Ga-DOTA-TOC, respectively.

#### Ex vivo biodistribution: tumour binding

**Fig. 5:**
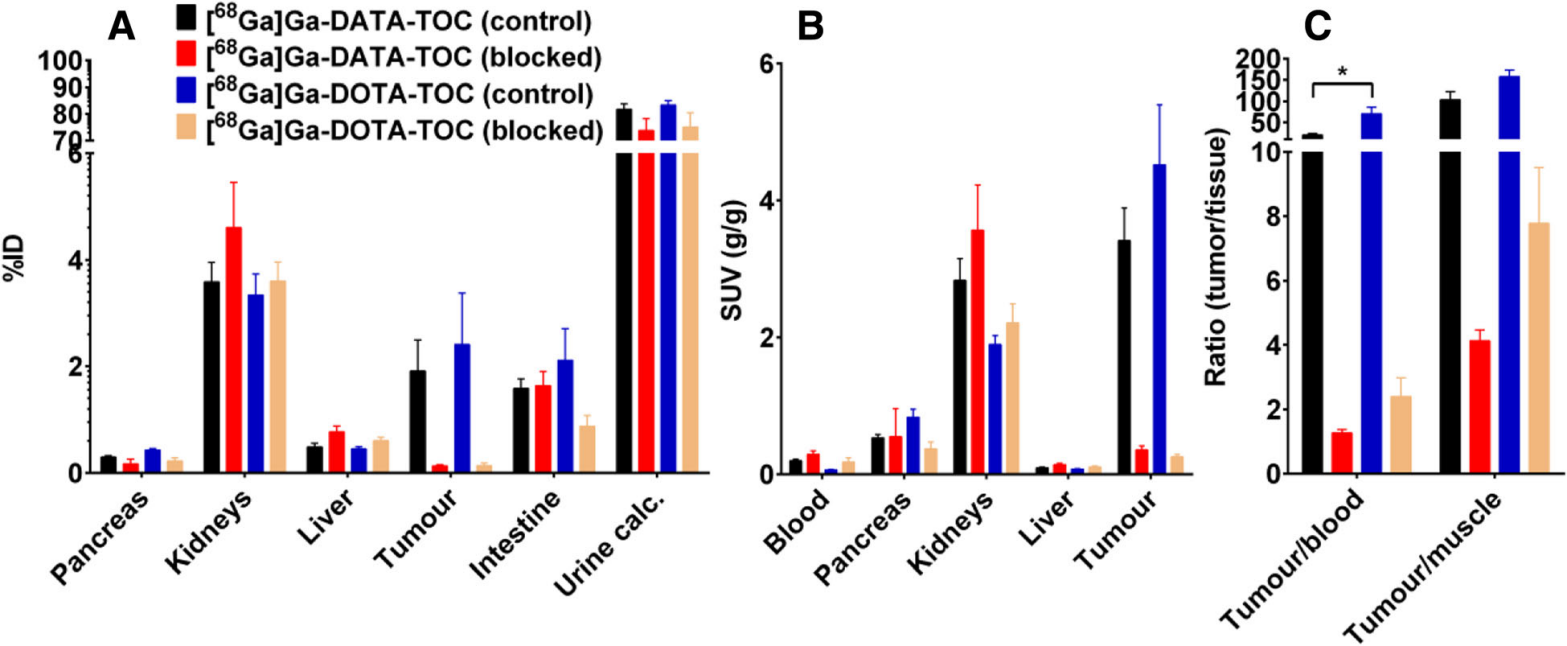
Biodistribution of [^68^Ga]Ga-DATA-TOC and [^68^Ga]Ga-DOTA-TOC in selected organs and tissues expressed as **a** percent of injected activity (%IA), **b** SUV in g/g, and **c** tumour-to-tissue ratios (SUV/SUV) 60 min after single intravenous injection of [^68^Ga]Ga-DATA-TOC (control *n* = 9, blocked *n* = 8) and [^68^Ga]Ga-DOTA-TOC (control *n* = 5, blocked *n* = 7) in MPC-mCherry tumour-bearing NMRI-nude mice; blocked animals received 100 μg/mouse [Nal^3^]octreotide acetate together with the radiotracer

Biodistributions of [^68^Ga]Ga-DATA-TOC and [^68^Ga]Ga-DOTA-TOC in subcutaneous MPC-mCherry tumour-bearing mice were analysed at 1 h p.i. (Fig. 5, tabular results are presented in the Additional file 1 in Tables S2 and S3) for quantitative comparison of tumour accumulation, distribution, and elimination in control and blocked state. Figure 5a shows values of uptake in terms of %ID, whilst Fig. 5b shows values in terms of SUV. Both graphs also show ratios derived from the results of the blocking studies.

**Fig. 6:**
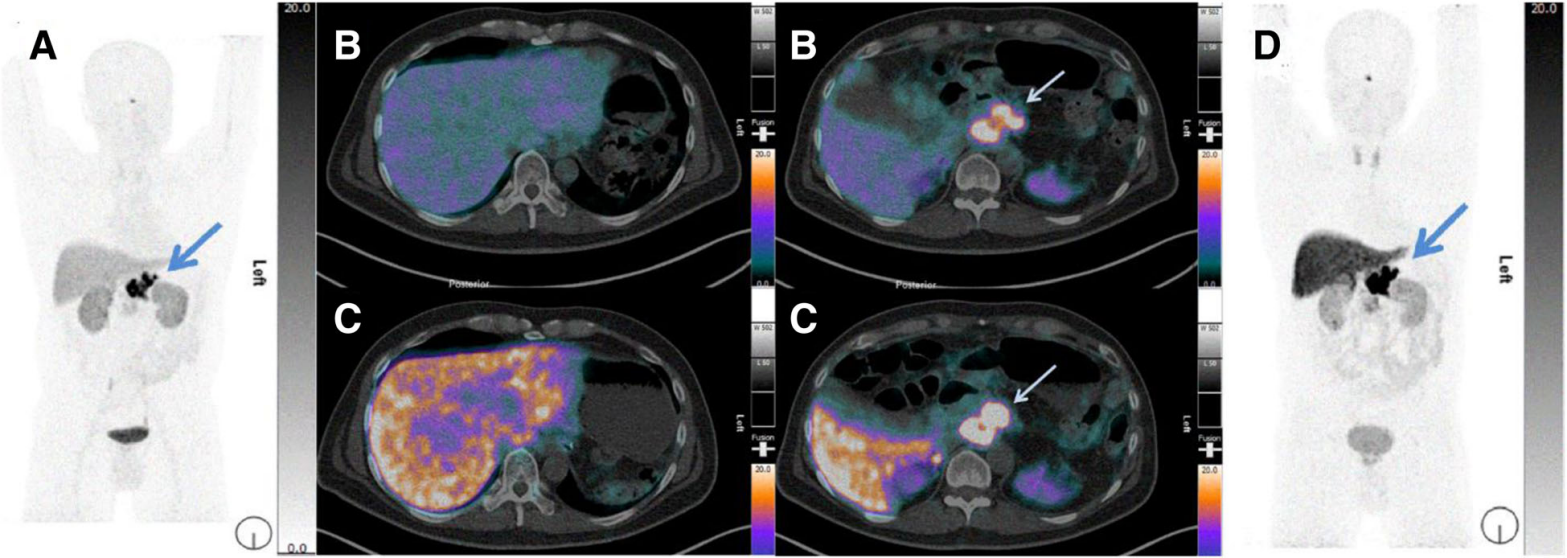
[^68^Ga]Ga-DATA-TOC and [^68^Ga] Ga-DOTATOC PET/CT images. **a** [^68^Ga]Ga-DATA-TOC PET MIP. **b** Transverse PET/CT fusion of [^68^Ga]Ga-DATA-TOC PET/CT images. **c** Transverse PET/CT fusion [^68^Ga]Ga-DOTA-TOC PET/CT images. **d** [^68^Ga]Ga-DOTA-TOC PET MIP. Arrows indicate high uptake in the primary pancreatic NET. The [^68^Ga]Ga-DATA-TOC PET/CT images show slightly higher physiological uptake in the kidneys as compared to the [^68^Ga]Ga-DOTA-TOC PET/CT study in the same patient. There is significantly higher uptake in normal liver after injection of [^68^Ga]Ga-DOTA-TOC in comparison with PET/CT images obtained after using [^68^Ga]Ga-DATA-TOC

The tumour uptake of [^68^Ga]Ga-DATA-TOC and [^68^Ga]Ga-DOTA-TOC at 1 h after injection was in the same range with SUVs of 3.41 ± 1.43 and 4.52 ± 1.96 (*P* = 0.2838), respectively. These quantitative and statistically relevant ex vivo data are consistent with the in vivo PET data shown in Fig. 3.

#### Ex vivo biodistribution: specific tumour binding

The simultaneous injection of excess [Nal^3^]octreotide clearly blocked the tumour accumulation for both radiotracers. The resulting activity concentrations were not statistically significantly different with 0.36 ± 0.17 SUV [^68^Ga]Ga-DATA-TOC and 0.26 ± 0.09 SUV [^68^Ga]Ga-DOTA-TOC, *P* = 0.2145.

#### Ex vivo biodistribution: binding to other tissues

*Blood and muscle*: The blood concentration of [^68^Ga]Ga-DATA-TOC (0.19 ± 0.08 SUV) was higher in comparison to [^68^Ga]Ga-DOTA-TOC (0.06 ± 0.01 SUV; *P* < 0.01). This resulted in a lower tumour-to-blood ratio of 20.2 ± 11.9 vs. 70.5 ± 34.3; *P* < 0.01. However, the tumour-to-muscle ratios for both radiotracers were not statistically different with 103.0 ± 57.2 for [^68^Ga]Ga-DATA-TOC and 157.0 ± 34.6 for [^68^Ga]Ga-DOTA-TOC (*P* = 0.1027). Ratios for tumour-to-blood and tumour-to-muscle at 1 h p.i. are graphically represented in Fig. 5c. This graph also shows ratios derived from the results of the blocking studies.

*Pancreas*: The pancreas expresses SST_2_ and was therefore investigated as well. Similar to the tumour, there was a higher uptake of [^68^Ga]Ga-DOTA-TOC in the pancreas with 0.84 ± 0.27 SUV compared to [^68^Ga]Ga-DATA-TOC with 0.53 ± 0.15 SUV, *P* < 0.05. The [Nal^3^]octreotide acetate injection decreased also the accumulation of the [^68^Ga]Ga-DOTA-TOC in the pancreas from 0.836 ± 0.267 to 0.374 ± 0.268 SUV, *P* < 0.05.

### Patient study

Compared with [^68^Ga]Ga-DOTA-TOC PET/CT before PRRT, post-PRRT [^68^Ga]Ga-DOTA-TOC PET/CT demonstrated partial disease remission according to molecular imaging criteria (65% decrease of uptake in the primary pancreatic tumour based on the target-to-pituitary ratio). PET/CT with [^68^Ga]Ga-DATA-TOC, performed 24 h later at the same time post-tracer injection, demonstrated a similar, very intense hSST_2_-uptake in the primary pancreatic tumour (Fig. 6). There was a notable lower uptake of ^68^Ga]Ga-DATA-TOC in normal liver (Table 1) compared to [^68^Ga]Ga-DOTA-TOC.

**Table 1:** Comparison of SUVs between [^68^Ga]Ga-DATA-TOC and [^68^Ga]Ga-DOTA-TOC in a 46-year-old male patient with a well-differentiated NET in the body and tail of the pancreas

Location	SUV	
	[^68^Ga]Ga-DATA-TOC	[^68^Ga]Ga-DOTA-TOC
Target lesion	46.9	71.1
Liver	9.1	23.1
Ratio (target-to-liver)	5.2	3.1
Blood	2.7	2.0
Ratio (target-to-blood)	17.4	35.6
Pituitary	14.6	23.7
Ratio (target-to-pituitary gland)	3.2	3.0

## Discussion

The novel TOC-conjugate, DATA-TOC, showed the potential to establish an instant kit-type labelling routine of clinically relevant vectors with ^68^Ga [23, 33]. To establish that the DATA chelator does not negatively affect the receptor affinity and the in vivo performance of the targeting vector, [^68/nat^Ga]Ga-DATA-TOC was directly compared to [^68/nat^Ga]Ga-DOTA-TOC in a series of in vitro and in vivo studies.

Radiolabelling with ^68^Ga for animal studies was completed quantitatively at 20 °C for DATA-TOC, whereas for DOTA-TOC a higher temperature was required to achieve comparable labelling efficiency. This finding corroborates previously reported radiochemical data for convenient and simple kit-type labelling of DATA-TOC with ^68^Ga [23].

[^nat^Ga]Ga-DATA-TOC and [^nat^Ga]Ga-DOTA-TOC showed high affinity for the hSST_2_. Although [^nat^Ga]Ga-DOTA-TOC displayed fivefold higher affinity than [^nat^Ga]Ga-DATA-TOC in this assay, absolute differences in the pertinent IC_50_ values were < nM (Fig. 3). Based on previous studies, such differences can be considered minimal for clinical translation given that many other critical factors (such as agonism or antagonism, stability, pharmacokinetics, or tumour residence times) greatly affect final clinical outcomes [28, 30, 34, 35]. Therefore, it is reasonable to conclude that the exchange of DOTA for the DATA chelator was well tolerated by the hSST_2_-target. Furthermore, first in vivo small animal PET studies comparing [^68^Ga]Ga-DATA-TOC to [^68^Ga]Ga-DOTA-TOC showed similar biodistribution and kinetic profiles. The uptake in the tumours was specific, reaching comparable values and following similar kinetics. The tumour accumulation of both radiotracers was blocked by [Nal^3^]octreotide acetate with similiar activity concentration, suggesting an SST_2_-specific process. Ex vivo organ distribution data was collected to mitigate misleading micro-PET data that can be affected by photon energies of ^68^Ga, partial volume, and spill-over effects. Biodistribution of [^68^Ga]Ga-DATA-TOC and [^68^Ga]Ga-DOTA-TOC in subcutaneous MPC-mCherry tumour-bearing mice was analysed in terms of %IA and in terms of SUV. The tumour uptake of both [^68^Ga]Ga-DATA-TOC and [^68^Ga]Ga-DOTA-TOC at 1 h after injection was in the same range with SUVs of 3.41 ± 1.43 and 4.52 ± 1.96 (*P* = 0.2838), respectively. The simultaneous injection of excess [Nal^3^]octreotide distinctly blocked the tumour accumulation of both radiotracers. These quantitative and statistically relevant ex vivo data are in accordance to the in vivo small animal PET data.

The first in human comparison of [^68^Ga]Ga-DATA-TOC and [^68^Ga]Ga-DOTA-TOC showed comparable uptake in the tumour lesions. The SUVmax of the liver in this patient on [^68^Ga]Ga-DOTA-TOC PET/CT was higher than the previous value reported (23.1 vs 12.8 ± 3.6) [36]. However, in the head-to-head comparison, uptake into the normal liver was significantly lower with [^68^Ga]Ga-DATA-TOC than with [^68^Ga]Ga-DOTA-TOC PET/CT (9.1 vs 23.1). Although [^68^Ga]Ga-DATA-TOC resulted in a lower overall tumour uptake (SUV 46.9), a significantly better tumour-to-liver ratio of 5.2 (compared to 3.1 for [^68^Ga]Ga-DOTA-TOC) could be achieved, which might enable better visualisation of liver metastases [37].

The present study aimed to identify whether the new chelator DATA influences the affinity and pharmacology of the DATA-conjugated radiopharmaceutical [^68^Ga]Ga-DATA-TOC relative to the industry standard [^68^Ga]Ga-DOTA-TOC. The radiotracers displayed similar characteristics in terms of in vitro affinity and in vitro internalisation to SST-positive cell lines, as well in terms of organ distribution and uptake kinetics. [^68^Ga]Ga-DATA-TOC displays high potential as a diagnostic agent in PET/CT, whilst its ease of preparation adds an important aspect to the daily routine of radiotracer production. Ease of preparation is an important advantage and applies also for some other new chelators for ^68^Ga. THP and NOPO are two such examples which have been conjugated to different octreotide derivatives, specifically Tyr^3^-octreotate (TATE) and NaI^3^-octreotide (NOC). [^68^Ga]Ga-THP-TATE was synthesised and compared with [^68^Ga]Ga-DOTA-TATE [38]. Head-to-head comparisons were performed in terms of in vivo micro-PET imaging and ex vivo biodistribution in Balb/c nu/nu mice bearing SST_2_-positive AR42J tumours. Tumour uptake at 1 h p.i. showed that the uptake of [^68^Ga]Ga-THP-TATE relative to [^68^Ga]Ga-DOTA-TATE was slightly lower (~ 20%), whilst kidney retention was significantly higher. Liver accumulation and blood retention were higher for [^68^Ga]Ga-THP-TATE. Tumour-to-liver ratios obtained from PET images were lower for [^68^Ga]Ga-THP-TATE (10.5) than for [^68^Ga]Ga-DOTA-TATE (27.2). The differences were, in part, attributed to significantly increased lipophilicity of [^68^Ga]Ga-THP-TATE (almost five times more lipophilic than [^68^Ga]Ga-DOTA-TATE). Indeed, a similar trend was reported for another [^68^Ga]Ga-THP-conjugated radiopharmaceutical based on the RGD vector, namely [^68^Ga]Ga-THP-NCS-RGD and [^68^Ga]Ga-THP-PhNCS-RGD [39]. NOPO was attached to the octreotide derivative NaI^3^-octreotide (NOC), labelled with ^68^Ga and evaluated in athymic CD-1 nude mice with AR42J tumours using micro-PET imaging and ex vivo biodistribution [40]. Uptake of [^68^Ga]Ga-NOPO-NOC in the tumours was high and specific, whilst uptake in other organs and tissue was low with the exception of the kidneys.

It is not surprising that for the same molecular targeting vector (e.g. octreotide) and the radionuclide (e.g. ^68^Ga), the chelate will make a difference to the characteristics of the resulting radiotracer. Therefore in the development of new radiotracers, it is critical to quantify and understand the impact any new chelate may have on binding affinity, internalisation, organ distribution, uptake kinetics, and excretion pathways of a certain type of radiopharmaceutical in head-to-head assays. This will demonstrate to what extent the ease of radiolabelling demonstrated for a new group of chelators can be translated into clinical application, challenging the state-of-the-art chelators such as DOTA and ultimately to the benefit of patients.

## Conclusion

It has been shown that [^68^Ga]Ga-DATA-TOC can be prepared in a simple kit-type manner, and under milder conditions than the DOTA-based counterpart. The described small animal studies and first-in-human study showed [^68^Ga]Ga-DATA-TOC equally able, and in some cases slightly better, for the visualisation of NET lesions compared to [^68^Ga]Ga-DOTA-TOC. Combining these results with the in vitro data, the chelator-switch from DOTA to DATA did not negatively affect the biological efficiency of the ^68^Ga-labelled TOC. Thus, this proof-of-principle study demonstrated the practical advantages of DATA for instant kit-type labelling without negatively affecting the efficacy.

These advantages highlight the potential of the DATA chelator as a promising tool for ^68^Ga-radiolabelling in general, but especially for radiolabelling of heat- and/or pH-sensitive vectors. As a future perspective, the instant-kit type labelling of DATA-based molecular vectors will be broadened to include other medically interesting molecules, such as antibody fragments.

## Additional files

Additional file 1: **Table S1.** Binding affinities of [^nat^Ga]Ga-DATA-TOC and [^nat^Ga]Ga-DOTA-TOC on hSST_2/3/5_, as determined during displacement of [^125^I-Tyr^25^]LTT-SS28 from transfected HEK293-hSST_2/3/5_ cell membranes; LTT-SS28 served as reference. **Table S2.** Uptake in terms of %IA of [^68^Ga]Ga-DATA-TOC or [^68^Ga]Ga-DOTA-TOC in selected organs of MPC-mCherry tumour-bearing female NMRI nu/nu mice 1 h p.i. (218 ± 57 MBq (11.2 nmol peptide/kg) and 441 ± 109 MBq (10.5 nmol peptide)/kg body weight, respectively; blocking after coinjection of 100 µg/mouse [Nal^3^]octreotide acetate)). **Table S3.** Radioactivity concentration in terms of SUV of [^68^Ga]Ga-DATA-TOC or [^68^Ga]Ga-DOTA-TOC in selected organs of MPC-mCherry tumour-bearing female NMRI nu/nu mice 1 h p.i. (218 ± 57 MBq (11.2 nmol peptide/kg) and 441 ± 109 MBq (10.5 nmol peptide)/kg body weight, respectively; blocking after coinjection of 100 µg/mouse [Nal^3^]octreotide acetate)). **Figure S1.** (A) Cyclic chelators used for ^68^Ga: DOTA, NOTA, TRAP and (B) acyclic chelators used for ^68^Ga: DFO, DTPA, HDEB, and a bifunctional version of THP. (DOCX 104 kb)

## Abbreviations

BFC: Bifunctional chelator
CT: Computed tomography
DATA: 6-Amino-1,4-diazapine-triacetate
DFO: Deferoxamine
DOTA: 1,4,7,10-Tetraazacyclododecane-1,4,7,10-tetraacetic acid
DTPA: Diethylenetriaminepentaacetic acid
HBED: *N*,*N*′-Bis(2-hydroxybenzyl)ethylenediamine-*N*,*N*′-diacetic acid
HEPES: 2-[4-(2-hydroxyethyl)piperazin-1-yl]ethanesulfonic acid
IC_50_: Half-maximal inhibitory concentration
LTT-SS28: (H-Ser-Ala-Asn-Ser-Asn-Pro-Ala-Leu-Ala-Pro-Arg-Glu-Arg-Lys-Ala-Gly-c[Cys-Lys-Asn-Phe-Phe-DTrp-Lys-Thr-Tyr-Thr-Ser-Cys]-OH)
MeCN: Acetonitrile
MIP: Maximum intensity projection
NET: Neuroendocrine tumour
NOTA: Triazacyclononane triacetic acid
PET: Positron emission tomography
PPRT: Peptide receptor radiation therapy
SEM: Standard error of mean
SST: Somatostatin subtype receptor
SUV: Standard uptake value
TATE: DPhe-c[Cys-Tyr-DTrp-Lys-Thr-Cys]-Thr-OH)
TFA: Triflouroacetic acid
THP: Tris(hydroxypyridinone)
TOC: [Tyr^3^]octreotide (DPhe-c[Cys-Tyr-DTrp-Lys-Thr-Cys]-Thr-ol)
TRAP: Triazacyclononane-phosphinic acid
