# Supplementary material for: Instant kit preparation of 68Ga-radiopharmaceuticals via the hybrid chelator DATA: clinical translation of [68Ga]Ga-DATA-TOC
Source: EJNMMI Res. 2019 May 23;9:48. doi: 10.1186/s13550-019-0516-7 (PMC6533321; doi:10.1186/s13550-019-0516-7)
Supplement: Supplementary file 1 — Table S1. Binding affinities of [natGa]Ga-DATA-TOC and [natGa]Ga-DOTA-TOC on hSST2/3/5, as determined during displacement of [125I-Tyr25]LTT-SS28 from transfected HEK293-hSST2/3/5 cell membranes; LTT-SS28 served as reference. Table S2. Uptake in terms of %IA of [68Ga]Ga-DATA-TOC or [68Ga]Ga-DOTA-TOC in selected organs of MPC-mCherry tumour-bearing female NMRI nu/nu mice 1 h p.i. (218 ± 57 MBq (11.2 nmol peptide/kg) and 441 ± 109 MBq (10.5 nmol peptide)/kg body weight, respectively; blocking after coinjection of 100 µg/mouse [Nal3]octreotide acetate)). Table S3. Radioactivity concentration in terms of SUV of [68Ga]Ga-DATA-TOC or [68Ga]Ga-DOTA-TOC in selected organs of MPC-mCherry tumour-bearing female NMRI nu/nu mice 1 h p.i. (218 ± 57 MBq (11.2 nmol peptide/kg) and 441 ± 109 MBq (10.5 nmol peptide)/kg body weight, respectively; blocking after coinjection of 100 µg/mouse [Nal3]octreotide acetate)). Figure S1. (A) Cyclic chelators used for 68Ga: DOTA, NOTA, TRAP and (B) acyclic chelators used for 68Ga: DFO, DTPA, HDEB, and a bifunctional version of THP. (DOCX 104 kb) [file 13550_2019_516_MOESM1_ESM.docx]

**Supporting Information**

**Instant kit preparation of ^68^Ga-radiopharmaceuticals via the chimeric chelator DATA:**

**clinical translation of [^68^Ga]Ga-DATA-TOC**

Jean-Philippe Sinnes^1^ **·** Johannes Nagel^1^ **·** Bradley Waldron^1^ **·** Theodosia Maina ^2^ **·** Berthold A. Nock^2^ **·** Ralf K. Bergmann^3^ **·** Martin Ullrich^3^ **·** Jens Pietzsch^3,4^ **·** Michael Bachmann^3,5,6^ **·** Richard P. Baum^7^ **·** Frank Rösch^1*^

**Author details:**

^1^Johannes Gutenberg-University Mainz, Institute of Nuclear Chemistry, Germany

^2^Molecular Radiopharmacy, INRASTES, NCSR "Demokritos", Athens, Greece

^3^Helmholtz-Zentrum Dresden-Rossendorf, Institute of Radiopharmaceutical Cancer Research, Dresden, Germany

^4^Technische Universität Dresden, School of Science, Faculty of Chemistry and Food Chemistry, Dresden, Germany

^5^Technische Universität Dresden, Universitätsklinikum ‘Carl Gustav Carus’, Universitäts-Krebs-Centrum (UCC), Tumorimmunology, Dresden, Germany

^6^Technische Universität Dresden, National Center for Tumor Diseases (NCT), Dresden, Germany

^7^Zentralklinik Bad Berka GmbH, Clinic for Molecular Radiotherapy, Bad Berka, Germany

**Table S1**.

Binding affinities of [^nat^Ga]Ga-DATA-TOC and [^nat^Ga]Ga-DOTA-TOC on hSST_2/3/5_, as determined during displacement of [^125^I-Tyr^25^]LTT-SS28 from transfected HEK293-hSST_2/3/5_ cell membranes; LTT-SS28 served as reference.

| **Compound** | **hSST_2_** | **hSST_3_** | **hSST_5_** |
| --- | --- | --- | --- |
| [^nat^Ga]Ga-DATA-TOC | 1.03±0.08 nM (3)* | -** | -** |
| [^nat^Ga]Ga-DOTA-TOC | 0.21±0.01 nM (2) | -** | -** |
| LTT-SS28 | 0.05±0.01 nM (3) | 0.09±0.01nM (3) | 0.17±0.03 nM (3) |

*IC_50_±sd in nM (number of independent assays performed in triplicate); **Not possible to determine due to excessive non-specific binding in the applied concentration range.

**Table S2**.

Uptake in terms of %IA of [^68^Ga]Ga-DATA-TOC or [^68^Ga]Ga-DOTA-TOC in selected organs of MPC-mCherry tumour-bearing female NMRI nu/nu mice 1 h p.i. (218 ± 57 MBq (11.2 nmol peptide/kg) and 441 ± 109 MBq (10.5 nmol peptide)/kg body weight, respectively; blocking after coinjection of 100 µg/mouse [Nal^3^]octreotide acetate)).
Values are presented as mean %IA ± SD with P-values from *post hoc* Tukey´s multiple comparisons test indicating statistical significance between two values when P < 0.05 (only values < 0.05 displayed):
P1 = comparison for control versus blocked [^68^Ga]Ga-DATA-TOC;
P2 = comparison for control versus blocked [^68^Ga]Ga-DOTA-TOC;
P3 = comparison between the [^68^Ga]Ga-DATA-TOC and the [^68^Ga]Ga-DOTA-TOC values for control;
P4 = comparison between the [^68^Ga]Ga-DATA-TOC and the [^68^Ga]Ga-DOTA-TOC values for blocked.

| %IA | [^68^Ga]Ga-DATA-TOC | | | [^68^Ga]Ga-DOTA-TOC | | |  | Comparison | |
| --- | --- | --- | --- | --- | --- | --- | --- | --- | --- |
| Type | Control n=9 | Blocked n=8 | P1 | Control n=7 | Blocked n=5 | P2 |  | P3 | P4 |
| Brain | 0.02±0.01 | 0.01±0.00 |  | 0.00±0.00 | 0.02±0.01 |  |  |  |  |
| Ovaries | 0.03±0.03 | 0.05±0.02 |  | 0.00±0.00 | 0.02±0.01 |  |  |  |  |
| Uterus | 0.17±0.31 | 0.13±0.08 |  | 0.02±0.00 | 0.08±0.02 |  |  |  |  |
| Pancreas | 0.29±0.07 | 0.16±0.26 |  | 0.42±0.06 | 0.22±0.16 |  |  | <0.05 |  |
| Spleen | 0.03±0.01 | 0.05±0.02 |  | 0.01±0.00 | 0.03±0.01 |  |  |  |  |
| Adrenal | 0.01±0.00 | 0.01±0.00 |  | 0.00±0.00 | 0.00±0.00 |  |  |  |  |
| Kidneys | 3.58±1.11 | 4.60±2.41 |  | 3.34±0.87 | 3.60±0.95 |  |  |  |  |
| Heart | 0.04±0.03 | 0.08±0.05 |  | 0.01±0.00 | 0.06±0.07 |  |  |  |  |
| Lung | 0.16±0.07 | 0.21±0.10 |  | 0.12±0.01 | 0.19±0.14 |  |  |  |  |
| Thyroid | 0.01±0.00 | 0.02±0.01 |  | 0.00±0.00 | 0.01±0.00 |  |  | <0.05 |  |
| Gall bladder | 0.00±0.01 | 0.02±0.03 |  | 0.00±0.00 | 0.00±0.00 |  |  |  |  |
| Liver | 0.48±0.21 | 0.77±0.30 | <0.05 | 0.45±0.09 | 0.60±0.16 |  |  |  |  |
| Femur | 0.04±0.01 | 0.06±0.03 |  | 0.04±0.04 | 0.04±0.02 |  |  |  |  |
| Tumour | 1.90±1.74 | 0.13±0.05 | <0.05 | 2.40±1.94 | 0.13±0.12 | <0.05 |  |  |  |
| Intestine | 1.58±0.53 | 1.63±0.75 |  | 2.11±1.33 | 0.87±0.49 |  |  |  |  |
| Stomach | 0.55±0.18 | 0.25±0.14 | <0.01 | 1.34±0.39 | 0.67±0.42 | <0.05 |  | <0.001 | <0.05 |
| Urine calc. | 81.7±6.26 | 73.7±12.6 |  | 83.3±3.73 | 75.0±14.2 |  |  |  |  |

**Table S3.**

Radioactivity concentration in terms of SUV of [^68^Ga]Ga-DATA-TOC or [^68^Ga]Ga-DOTA-TOC in selected organs of MPC-mCherry tumour-bearing female NMRI nu/nu mice 1 h p.i. (218 ± 57 MBq (11.2 nmol peptide/kg) and 441 ± 109 MBq (10.5 nmol peptide)/kg body weight, respectively; blocking after coinjection of 100 µg/mouse [Nal^3^]octreotide acetate)).
Values are presented as mean SUV ± SD with P-values from *post hoc* Tukey´s multiple comparisons test indicating statistical significance between two values when P < 0.05 (only values < 0.05 displayed):
P1 = comparison for control versus blocked [^68^Ga]Ga-DATA-TOC;
P2 = comparison for control versus blocked [^68^Ga]Ga-DOTA-TOC;
P3 = comparison between the [^68^Ga]Ga-DATA-TOC and the [^68^Ga]Ga-DOTA-TOC values for control;
P4 = comparison between the [^68^Ga]Ga-DATA-TOC and the [^68^Ga]Ga-DOTA-TOC values for blocked.

| SUV | [^68^Ga]Ga-DATA-TOC | | | [^68^Ga]Ga-DOTA-TOC | | | Comparison | |
| --- | --- | --- | --- | --- | --- | --- | --- | --- |
| Type | Control n=9 | Blocked n=8 | P1 | Control n=7 | Blocked n=5 | P2 | P3 | P4 |
| Blood | 0.19±0.08 | 0.29±0.14 |  | 0.06±0.01 | 0.17±0.16 |  | <0.01 |  |
| BAT | 0.08±0.04 | 0.15±0.08 |  | 0.03±0.01 | 0.10±0.06 |  |  |  |
| Skin | 0.19±0.13 | 0.31±0.17 |  | 0.09±0.01 | 0.21±0.12 |  |  |  |
| Brain | 0.01±0.01 | 0.01±0.00 |  | 0.00±0.00 | 0.01±0.00 |  |  |  |
| Ovaries | 0.16±0.13 | 0.20±0.11 |  | 0.03±0 | 0.13±0.22 |  |  |  |
| Uterus | 0.24±0.36 | 0.18±0.07 |  | 0.05±0 | 0.21±0.15 |  |  |  |
| Pancreas | 0.53±0.15 | 0.55±1.16 |  | 0.83±0.26 | 0.37±0.26 | <0.05 | <0.05 |  |
| Spleen | 0.06±0.02 | 0.11±0.03 | <0.05 | 0.04±0.01 | 0.06±0.03 |  |  |  |
| Adrenals | 0.19±0.14 | 0.19±0.07 |  | 0.09±0.10 | 0.16±0.18 |  |  |  |
| Kidneys | 2.83±0.95 | 3.56±1.87 |  | 1.89±0.29 | 2.21±0.74 |  |  |  |
| WAT | 0.04±0.14 | 0.38±0.72 |  | 0.04±0.02 | 0.06±0.02 |  |  |  |
| Muscle | 0.04±0.04 | 0.09±0.05 |  | 0.03±0.01 | 0.05±0.05 |  |  |  |
| Heart | 0.09±0.05 | 0.14±0.09 |  | 0.03±0.00 | 0.09±0.11 |  |  |  |
| Lung | 0.20±0.08 | 0.29±0.15 |  | 0.16±0.03 | 0.22±0.13 |  |  |  |
| Liver | 0.09±0.04 | 0.14±0.05 |  | 0.07±0.01 | 0.10±0.04 |  |  |  |
| Femur | 0.06±0.02 | 0.12±0.06 |  | 0.06±0.04 | 0.09±0.05 |  |  |  |
| Tumour | 3.41±1.43 | 0.35±0.16 | <0.001 | 4.52±1.96 | 0.25±0.09 | <0.001 |  |  |
| Tumour/blood | 20.2±11.9 | 1.26±0.30 | <0.001 | 70.5±34.3 | 2.38±1.55 | <0.001 | <0.01 |  |
| Tumour/muscle | 103.±57.2 | 4.12±0.96 | <0.001 | 157.±34.6 | 7.77±4.61 | <0.001 |  |  |

FIGURE S1 (A) Cyclic chelators used for ^68^Ga: DOTA, NOTA, TRAP and (B) acyclic chelators used for ^68^Ga: DFO, DTPA, HDEB, and a bifunctional version of THP
